# Aetiology of sepsis in adults living with HIV in East Africa: a secondary analysis of an open-label, multicentre, randomised, controlled phase 3 trial

**DOI:** 10.1016/j.eclinm.2025.103719

**Published:** 2026-01-28

**Authors:** Eva Otoupalova, Lucas Ampaire, Megan Null, Buliga Mujaga, Jie Liu, Bibie Said, Edwin Nuwagira, Margaretha Sariko, Geofrey Gidoi, Antony Gulinja, Samuel Jjunju, Arestaricky Rimoy, Fredrick C. Mwita, Jeremiah Kidola, Oscar Atwiine, David Ocaaki, Dalton K. Munyambalu, Prakruti Rao, David R. Boulware, Tania A. Thomas, Stellah Mpagama, Conrad Muzoora, Scott K. Heysell, Christopher C. Moore

**Affiliations:** a Division of Pulmonary, Critical Care, and Environmental Medicine, Department of Medicine, Tulane University School of Medicine, New Orleans, USA; b Department of Medical Laboratory Sciences, Mbarara University School of Science and Technology Faculty of Medicine, Mbarara, Uganda; c Division of Infectious Diseases and International Health, Department of Medicine, University of Virginia School of Medicine, Charlottesville, USA; d Biotechnology Laboratory, Kilimanjaro Clinical Research Institute, Moshi, Tanzania; e Department of Medicine, Kibong'oto Infectious Disease Hospital, Sanya Juu, Tanzania; f Department of Medicine, Mbarara University School of Science and Technology Faculty of Medicine, Mbarara, Uganda; g Mwanza Research Centre, National Institute for Medical Research, Mwanza, Tanzania; h Fort Portal Regional Referral Hospital, Fort Portal, Uganda; i Division of Infectious Diseases and International Medicine, Department of Medicine, University of Minnesota, Minneapolis, USA

**Keywords:** Tuberculosis, HIV, Sepsis, Africa

## Abstract

**Background:**

Sepsis in people living with HIV (PLWH) in East Africa has high mortality. Regionally, the etiology of sepsis is incompletely understood. We performed a planned analysis of the microbiological data obtained from a randomised clinical trial of early empiric anti-*Mycobacterium tuberculosis* (*Mtb*) therapy for sepsis (ATLAS) in Tanzania and Uganda.

**Methods:**

We present a prespecified, secondary analysis of a phase three, open-label, multicentre, randomised, controlled trial conducted at four regional referral hospitals in Tanzania and Uganda. Participants were adults living with HIV admitted with concern for infection and a modified quick sepsis-related organ failure assessment (qSOFA) ≥2. Participants were randomised to (1) immediate or diagnosis-dependent antituberculosis therapy and to (2) high-dose or conventional-dose antituberculosis therapy. Tests for sepsis etiology included bacterial blood and urine cultures, multi-pathogen qPCR from blood, GeneXpert MTB/RIF Ultra from sputum and urine, urine lipoarabinomannan (LF-LAM), and *Mtb* cultures from sputum and blood. We used multivariable logistic regression analysis and random forest analysis to determine variables that predicted *Mtb* as the sepsis etiology. The trial is registered with ClinicalTrials.gov, NCT04618198.

**Findings:**

From January 5, 2022 through December 9, 2024, we randomised 437 participants to receive immediate and/or high dose antituberculosis therapy. *Mtb* was the most common pathogen, detected in 229 (52%) of 437 participants, and in 54 (50%) of 108 participants with a bloodstream infection. Combined urine LF-LAM and sputum GeneXpert MTB/RIF testing missed 17 (32%) of 54 *Mtb* bloodstream infections. The most frequent non-mycobacterial bacteria were *Klebsiella* species and *Escherichia coli*, which were identified in 39 (9%) and 33 (8%) of 437 participants, respectively. We detected ceftriaxone resistance in 21 (64%) of 33 bacterial isolates. In a random forest prediction model (accuracy: 0.6; precision: 0.5; recall: 0.6; F1-score: 0.5), the best indicators of *Mtb* as a sepsis pathogen were a greater number of ill-days before presentation (mean decrease in accuracy [MDA] 10.1), younger age (MDA 8.7), a longer duration of cough (MDA 7.7), and low CD4+ T-cell concentration (MDA 3.7).

**Interpretation:**

*Mtb* was the most common pathogen causing sepsis and bloodstream infection and was frequently missed by conventional rapid diagnostics. We also identified a high prevalence of non-mycobacterial pathogens resistant to ceftriaxone in blood and urine cultures. Limitations of our study included exclusion of cryptococcal antigen positive participants, non-systematic drug susceptibility testing, and potential regional differences in sepsis etiology and resistance patterns.

**Funding:**

NIH.

**Research in context**

**Evidence before this study**

We searched PubMed, Embase, and MEDLINE for studies investigating sepsis etiology in low- and middle-income countries (LMICs) from database inception up to July 29, 2025, with the terms “sepsis”, “etiology” and “Africa”. Of the 20 studies that we identified from Africa that examined sepsis etiology, 11 were performed in adults, and out of those, most studies included a subgroup of PLWH. The most frequent cause of HIV-associated sepsis in Africa was *Mtb* infection (9–27%) followed by *Streptococcus pneumoniae* (9%–15%). Although *Mtb* was the most frequently isolated pathogen, only a small number of studies specifically assessed for *Mtb* bacteremia. Furthermore, only one study performed WHO-recommended rapid tuberculosis (TB) testing with urine lateral flow lipoarabinomannan antigen (LF-LAM) and sputum nuclear acid amplification tests on all enrolled participants. Additionally, most of the studies were only able to identify a small proportion of causative sepsis pathogens, and only three studies included blood or tissue PCR to evaluate for less common bacterial and viral pathogens. Therefore, the underlying microbiology of sepsis is in PLWH in Africa is unclear, and the optimal diagnostic and treatment strategies remain unknown.

**Added value of this study**

In this study, we identified *Mtb* in 229 (52%) of 437 study participants presenting with sepsis, which was a higher proportion than previous studies given our comprehensive testing. We found that combined urine LF-LAM and sputum GeneXpert MTB/RIF testing missed 32% of *Mtb* bloodstream infections and identified a high prevalence of ceftriaxone resistance among non-*Mtb* bacterial isolates. These findings highlight the limitations of current WHO tuberculosis diagnostics, the need to evaluate test performance in HIV-related sepsis, and the importance of developing evidence-based, pathogen-specific empiric antibiotic strategies in high HIV/tuberculosis prevalence settings.

**Implications of all the available evidence**

Our study, in combination with previous literature, suggests that empirical regimens for PLWH with sepsis in tuberculosis endemic regions should include treatment of *Mtb* and account for other ceftriaxone-resistant bacteria. Our study is a pre-specified secondary analysis of a clinical trial and as such was not designed for assessing drug resistance; however, the detected resistance patterns agree with reported rising rates of cephalosporin resistance in the African continent.

## Introduction

Sepsis is a syndrome of dysregulated host response to infection leading to organ dysfunction and has a high associated mortality.^1^^,^^2^ The annual incidence of sepsis is estimated at 48 million cases worldwide, and 85% of cases occur in low- and middle-income countries (LMICs). Despite advances in sepsis diagnostics and treatment, sepsis continues to cause approximately 11 million deaths globally every year, most of which are preventable.^3^ Despite the disproportionate burden of sepsis in LMICs, the data that support recommendations for sepsis management come overwhelmingly from high-income countries (HICs). Furthermore, some treatments adopted from HICs, such as liberal administration of fluids to patients presenting with sepsis, are harmful to patients in LMICs.^4^^,^^5^

With case fatality rates as high as 39%, Africa is one of the regions most affected by sepsis.^6^ The reasons are likely multifactorial and include the relative lack of high-quality studies on sepsis etiology and antimicrobial resistance. Importantly, most sepsis-related deaths in Africa occur among people living with human immunodeficiency virus (PLWH).^7^ Sepsis in PLWH can be caused by a wide spectrum of pathogens including *Mycobacterium tuberculosis* (*Mtb*), other non-*Mtb* bacteria, fungi, viruses, and other co-infections. We and others have shown in observational studies that 37–55% of PLWH with sepsis in East Africa tested positive for *Mtb*, and a third were co-infected with another pathogen.^8^^,^^9^ Despite the high prevalence of *Mtb* sepsis, few studies from Africa have focused on *Mtb* detection from blood and have instead used urine diagnostics such as lateral flow testing for lipoarabinomannan (LF-LAM) or sputum-based diagnostics such as GeneXpert MTB/RIF or GeneXpert MTB/RIF Ultra. Sputum testing may lead to an underestimation of *Mtb* as a causative pathogen in PLWH with sepsis in Africa given the paucibacillary nature and extrapulmonary dissemination of tuberculosis among PLWH, and the difficulty with expectoration among the critically ill.

Delayed diagnosis and inappropriate initial antibiotic selection correlate with increased sepsis mortality.^10^ Current antimicrobial treatment for sepsis in most regions of Africa consists of a third-generation cephalosporin, which is typically ceftriaxone. Per World Health Organization guidelines, tuberculosis treatment is initiated only after a positive result from a rapid diagnostic test, or when clinical deterioration occurs despite several days of standard antibiotic therapy.^11^ It is therefore imperative to develop regionally appropriate standard diagnostic algorithms and antibiotic regimens for PLWH with sepsis that more accurately reflect the underlying sepsis etiology. Since inappropriate antibiotic therapy is associated with an increased risk of death from sepsis, understanding regional microbiology is key to improving sepsis survival. However, a significant knowledge gap regarding sepsis etiology in regions of Africa, especially among PLWH, still exists. Therefore, we performed an analysis of comprehensive microbiological testing of blood, urine, and sputum from all participants enrolled in a multicenter randomised controlled trial of immediate and/or high dose antituberculosis therapy compared to standard therapy in PLWH presenting with sepsis in Tanzania and Uganda.^12^

## Methods

### Study design, settings, and participants

This study is a prespecified, secondary analysis of a randomised clinical trial of early empiric anti-*M. tuberculosis* therapy for sepsis in sub-Saharan Africa (ATLAS trial).^12^ ATLAS trial was a multicenter, phase three, open label, randomised, 2 × 2 factorial trial to evaluate empiric early and/or high dose anti-TB therapy versus standard antibiotic therapy in participants admitted with clinical concern for sepsis to the regional referral hospitals Kibong'oto Infectious Diseases Hospital and Sekou Toure Regional Referral Hospital in Tanzania, and the Mbarara Regional Referral Hospital and Fort Portal Regional Referral Hospital in Uganda (NCT 04618198). We enrolled participants if they were ≥18 years old, living with HIV in Tanzania or Uganda, and were admitted to one of the study hospitals with presumed sepsis. We defined sepsis as clinical concern for infection and a modified quick sepsis-related organ failure assessment (qSOFA) ≥2 of the following: Glasgow coma scale score <15, a respiratory rate ≥22 breaths per minute, a systolic blood pressure ≤90 mmHg, or a mean arterial pressure of ≤65 mmHg. We excluded potential participants if they were treated with antibiotics at another inpatient facility for over 24 h prior to screening, were receiving or had received antituberculosis therapy in the last year, were pregnant, or had known liver disease or end-stage renal disease. We excluded potential participants with a positive whole blood cryptococcal antigen (CrAg) test from further testing and treatment due to their high case fatality rates, which would have biased the trial results. We collected clinical and laboratory data from each participant. Participants were randomised to (1) immediate or diagnosis-dependent antituberculosis therapy, and (2) high-dose rifampicin (30 mg/kg) and isoniazid (7.5 mg/kg) with conventional-dose ethambutol and pyrazinamide, or conventional-dose four-drug antituberculosis therapy for 28 days. Each patient also received ceftriaxone for 7 days. Randomisation was performed with computer-generated permuted-block algorithm which randomly assigned participants with equal probability to receive either immediate or diagnosis-dependent antituberculosis therapy and either high-dose or conventional WHO-recommended weight-based dose antituberculosis therapy. Only the trial coordinator and statistician were aware of mortality outcomes before study completion. All analyses presented in the manuscript were included as part of the prespecified secondary outcome of final sepsis etiology. The ATLAS trial received approval from the University of Virginia (HSR200253), the Tanzania Medicines and Medical Devices Authority (TMDA-WEB0021/CTR/0008/03) and National Institute for Medical Research (NIMR/HQ/R.8a/Vol. IX/3554), and the Uganda National Council for Science and Technology (HS1272ES). Informed consent was obtained from all individual participants included in the study, or, if unable to consent, from their caregiver or next of kin. The trial was performed according to published trial protocol.^12^^,^^13^ Sex at birth was obtained by self-report. As the study did not investigate identity or other psychosocial or cultural factors, gender was not assessed. The trial was conducted in accordance with the Consolidated Standards of Reporting Trials (CONSORT) guidelines to ensure transparency and completeness in reporting randomized controlled trials.

### Pathogen testing

Each participant underwent testing for sepsis etiology with blood (BacTec 9050) and urine culture. For evaluation of tuberculosis, all participants able to produce specimens underwent WHO-approved rapid tuberculosis diagnostic testing including GeneXpert MTB/RIF or GeneXpert MTB/RIF Ultra on sputum and urine, Alere Determine LF-LAM on urine, as well as mycobacterial culture of sputum (MGIT 960 system) and blood (BACTEC Myco/F system). Additionally, we tested blood with the multiplex qPCR TaqMan Array Card (TAC) assay, which targeted 43 pathogens (Supplementary Table S1) associated with febrile illness in East Africa, in batch analysis as previously described.^14^ We defined a positive target in the TAC assay as a cycle threshold <35. We tested for malaria parasites with the qPCR TaqMan Array Card (TAC) assay.

We defined tuberculosis as one or more positive tuberculosis test results. We defined a sepsis pathogen as any pathogen detected in urine or blood except for cytomegalovirus (CMV) and *Plasmodium* species, as they uncommonly cause sepsis in adults in East Africa,^15^^,^^16^ and incidental parasitemia or CMV antigenemia can occur asymptomatically in people with other critical illness. We defined bloodstream infection as a positive *Mtb* or non-*Mtb* bacterial blood culture, or a positive blood TAC assay for other causative pathogens except for CMV or *Plasmodium* species. We classified coagulase-negative staphylococci, *Micrococcus*, or further unidentifiable anaerobic bacterial growth as culture contaminants. The ATLAS trial did not include mandatory drug sensitivity testing; however, it was performed at the treating physician's discretion per local practice.

### Statistical analysis

We used descriptive statistics to calculate the distribution of non-*Mtb* bacterial, viral, fungal, protozoal, and *Mtb* infections and bloodstream infections in our cohort. We performed all descriptive analyses with simple frequencies. We used GraphPad Prism (version 10.2.3) for data analysis and data visualization. We created proportional Venn diagrams to determine the percentage of co-infections using the DeepVenn application,^17^ and non-proportional Venn diagrams using the online tool https://bioinformatics.psb.ugent.be/cgi-bin/liste/Venn. We created UpSet plots to determine the rates of TB tests co-positivity using the “Upset” package in RStudio (Version 2024.09.0+375).

We determined the most important variables predicting *Mtb* as a causative organism of sepsis through multivariable logistic regression analysis and a random forest algorithm conducted with the “randomForest” package in RStudio.^18^ Predictors included age, sex, weight, past *Mtb* disease, duration of illness before hospitalization, days of cough, current antiretroviral treatment, CD4+ T-cell concentration, and detectable HIV viral load. For the random forest model, we performed imputation for variables missing data and we generated the final dataset after ten iterations using the rfImpute function from the “randomForest” package. We stratified the sample into training (67%) and validation (33%) datasets, then performed Bootstrap sampling to generate multiple training datasets of the same total sample size for improved model robustness and to reduce overfitting. We calculated the mean decrease in accuracy, which measures the individual contribution of each variable, and the mean decrease Gini, to rank the importance of variables. We considered the remaining 33% of the dataset, which was not part of the bootstrap sample, to be out-of-bag samples. We determined the out-of-bag error rate, which indicated the model performance, through the “caret” package.^19^ Finally, we calculated accuracy, precision, recall, and F1-scores. Missing data for variables studied in random forest model was approximately 10%.

To determine to what degree an expanded antimicrobial regimen could improve antimicrobial coverage of sepsis in PLWH, we calculated the proportion of patients with *Mtb* that was missed by conventional WHO recommended GeneXpert MTB/RIF or GeneXpert MTB/RIF Ultra, and urine LF-LAM, and the proportion of pathogens that would not be expected to be treated with ceftriaxone alone.

### Role of the funding source

The funder of the study had no role in study design, data collection, data analysis, data interpretation, or writing of the report. The authors had full access to the data in the study. EO and CCM had final responsibility for the decision to submit for publication.

## Results

### Baseline characteristics

The ATLAS trial screened 707 participants for eligibility. We excluded 30 (4%) for positive cryptococcal antigen test and 240 (34%) for other reasons. Between January 5, 2022 and December 9, 2024, we randomised 437 participants. After late exclusions due to kidney or liver injury, we included 395 participants in the analysis of the primary 28-day mortality outcome; however, we included all 437 randomised participants in our secondary microbiology analysis (Supplementary Figure S1). A cryptococcal antigen test was positive in 30 (4%) of 707 screened potential participants, while TAC detected Cryptococcus in 1 (<1%) of 437 randomised participants. The median (interquartile range [IQR]) participant age was 42 (33–51) and 233 (53%) of 437 participants were female (Table 1). All participants were PLWH with a median (IQR) CD4+ T-cell concentration of 120 (29–332) and 244 (73%) of 337 tested patients had a detectable HIV viral load. Of the 437 randomised participants, 209 (48%) were receiving antiretroviral at the time of presentation. Those not already receiving antiretroviral therapy initiated it after admission to hospital per standard of care and treating physician discretion. The median (IQR) qSOFA score and Universal Vital Assessment (UVA) score were 2 (2) and 4 (4–6), respectively.^20^

**Table 1:** Baseline characteristics of participants in a randomized clinical trial of early empiric anti-*Mycobacterium tuberculosis* therapy for sepsis in sub-Saharan Africa (ATLAS trial).

Variable	Value
Age, years, median [IQR]	42 [33–51]
Female sex, n participants (%)	233 (53)
ART on admission, n participants (%)	209 (48)
Baseline CD4 count, cells/mm^3^, median [IQR]	120 [29–332]
Viral load, detectable, n participants (%)	244 (73)
Duration of illness prior to admission, days [IQR]	29 [14–60]
History of TB treatment prior to admission, n participants (%)	38 (9)
Systolic blood pressure mmHg, median [IQR]	88 [82–90]
Heart rate, beats/min, median [IQR]	120 [104–127]
Respiratory rate, beats/min, median [IQR]	26 [24–30]
Oxygen saturation, %spO 2, median [IQR]	95 [92–98]
qSOFA score [IQR]	2 [2]
UVA score [IQR]	4 [4–6]

All values rounded to integer except frequencies lower than 1. ART = anti-retroviral therapy; qSOFA score = modified quick sequential organ failure assessment score; UVA score = universal vital assessment score.

### Sepsis etiology

**Fig. 1:**
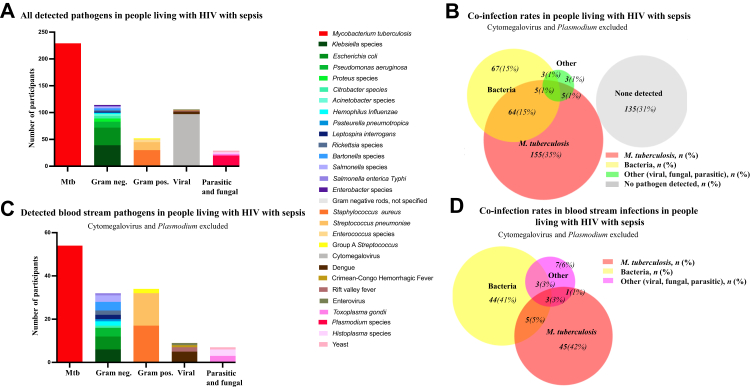
**Sepsis etiology in sub-Saharan Africa**. (A) Bar graph showing number of participants with detected pathogen. (C) Bar graph showing number of participants with detected pathogen bloodstream infection. Cytomegalovirus, *Cryptococcus* and *Plasmodium* were excluded. (B) Proportional Venn Diagram showing rates of co-infections in the study (n = number of participants; % percent of all participants included). (D) Proportional Venn Diagram showing rates of blood stream co-infections (n = number of participants; % percent of all participants with bloodstream infection); Cytomegalovirus, *Cryptococcus* and *Plasmodium* were excluded.

Of the 437 randomised participants, 302 (69%) had a detectable sepsis pathogen, 31 (7%) from blood alone, 146 (33%) from urine alone, 11 (3%) from sputum alone, and 77 (18%) from blood and another source (Supplementary Figure S2). We detected *Mtb* from one or more specimens in 229 (52%) participants*,* bacterial pathogens in 139 (32%) participants and other non-CMV viral, fungal, and non-*Plasmodium* parasitic pathogens in 16 (4%) participants (Fig. 1A and B and Table 2). We did not detect any non-tuberculous mycobacteria. Co-infection with non-CMV and non-*Plasmodium* organisms occurred in 77 (18%) of 437 participants (Fig. 1B). Of the 229 participants with tuberculosis identified by any test, 74 (17%) had a co-infection other than *Plasmodium* or CMV. CMV was detected concomitantly with 55 (24%) of 229 *Mtb* infections and 31 (22%) of 139 non-tuberculosis bacterial infections (Supplementary Figure S3A). *Plasmodium* was detectable with 6 (3%) of 229 *Mtb* infections and 14 (10%) of 139 bacterial infections (Supplementary Figure S3B).

**Table 2:** Microbiology of all detected organisms from participants in a randomized clinical trial of early empiric anti-*Mycobacterium tuberculosis* therapy for sepsis in sub-Saharan Africa (ATLAS trial).

Organism	Blood (n isolates, positive)	Urine (n isolates, positive)	Sputum (n isolates, positive)	Total (n, participants)	Total (% of all participants)
***Mycobacterium tuberculosis***	54	200	49	229	52.4
**Gram positive bacteria**					
*Staphylococcus aureus*	17	17		30	6.7
*Streptococcus pneumoniae*	15			15	3.4
*Enterococcus* species		5		5	1.1
Group A *Streptococcus*	2			2	0.5
**Gram negative bacteria**					
*Klebsiella* species	7	33		39	8.9
*Escherichia coli*	6	28		33	7.6
*Pseudomonas aeruginosa*	4	7		11	2.5
*Proteus* species		6		6	1.4
*Citrobacter* species	1	4		5	1.1
*Bartonella* species	4			4	0.9
*Salmonella* species	3			3	0.7
*Acinetobacter* species		3		3	0.7
*Coxiella burnetii*	2			2	0.5
*Haemophilus influenzae*	2			2	0.5
*Leptospira interrogans*	2			2	0.5
*Rickettsia* species	2			2	0.5
*Enterobacter* species		2		2	0.5
*Salmonella enterica Typhi*	1			1	0.2
*Pasteurella Pneumotropica*	1			1	0.2
Gram negative rods, unspecified		1		1	0.2
**Viruses**					
Cytomegalovirus	96			96	22.0
Dengue	5			5	1.1
Rift Valley Fever	2			2	0.5
Crimean Congo Hemorrhagic Fever	1			1	0.2
Enterovirus	1			1	0.2
**Fungi and parasites**					
*Plasmodium* species	20			20	4.6
Yeast	1	2		3	0.7
*Histoplasma* species	3			3	0.7
*Toxoplasma gondii*	3			3	0.7
*Cryptococcus neoformans*^a^	31^a^			31^a^	NA^a^

a 31 out of 707 screened participants were positive for cryptococcus (30 with positive CrAg, 1 with TAC). Participants with positive CrAg were excluded from the trial, therefore % positive participants is not available.

Of 108 participants with a bloodstream infection, we detected *Mtb,* non-*Mtb* bacterial pathogens, or other pathogens in 54 (50%), 55 (51%), and 14 (13%) participants, respectively (Fig. 1C and D and Table 3). Of the 54 *Mtb* positive test results from blood samples, we detected 45 (83%) by mycobacterial blood culture only, 5 (9%) from blood culture and qPCR TAC assay, and 9 (17%) from qPCR TAC assay only. Of the 55 cases of non-*Mtb* bacterial bloodstream infection, we detected 12 (22%) by bacterial blood culture and 45 (82%) by TAC (Table 3). Seven participants tested positive for more than one non-*Mtb* bacterial pathogen in blood. *Staphylococcus aureus* and *S. pneumoniae* were the most common non-*Mtb* bacterial bloodstream pathogens*,* followed by *Klebsiella* species, *Escherichia coli, Pseudomonas aeruginosa* and *Bartonella* species (Fig. 1C and Table 3). We also detected Group A *Streptococcus*, *Salmonella, Citrobacter*, *Coxiella burnetii* and *Leptospira* by TAC and in culture. Additionally, we detected several non-bacterial pathogens including Dengue*,* Rift Valley Fever*,* Enterovirus, *Histoplasma*, and *Toxoplasma* in blood by TAC*. Klebsiella* species, *E. coli, and S. aureus* were the most frequently isolated urinary pathogens. The prevalence of *Mtb* sepsis in Tanzania was 60% compared to 43% in Uganda (odds ratio [OR] 2.01, 95% confidence interval [CI] 1.37–2.95, p = 0.0004) (Supplementary Table S2).

**Table 3:** Bloodstream infection etiology in participants in a randomized clinical trial of early empiric anti-*Mycobacterium tuberculosis* therapy for sepsis in sub-Saharan Africa (ATLAS trial).

Organism	Culture (*n*, positive isolates)	TaqMan Array (*n*, positive isolates)	Total (*n*, patients)	Total (% of patients with blood stream infection)
***Mycobacterium tuberculosis***	45	14	54	42
**Gram positive bacteria**				
*Staphylococcus aureus*	4	13	17	16
*Streptococcus pneumoniae*	2	13	15	14
Group A *Streptococcus*	0	2	2	2
**Gram negative bacteria**				
*Escherichia coli*	0	6	6	6
*Klebsiella* species	1	6	6	6
*Pseudomonas aeruginosa*	3	1	4	4
*Bartonella* species	0	4	4	4
*Salmonella* species	1	2	3	3
*Coxiella burnetii*	0	2	2	2
*Haemophilus influenzae*	0	2	2	2
*Leptospira interrogans*	0	2	2	2
*Rickettsia* species	0	2	2	2
*Citrobacter* species	1	0	1	1
*Salmonella enterica Typhi*	0	1	1	1
*Pasteurella pneumotropica*	0	0	1	1
**Viruses**				
Dengue		5	5	5
Rift Valley Fever		2	2	2
Crimean Congo Haemmorhagic Fever		1	1	1
Enterovirus		1	1	1
**Fungi and parasites**				
Yeast		1	1	1
*Histoplasma* species		3	3	3
*Toxoplasma gondii*		3	3	3

All frequencies rounded to integer.

### Bacterial resistance

**Fig. 2:**
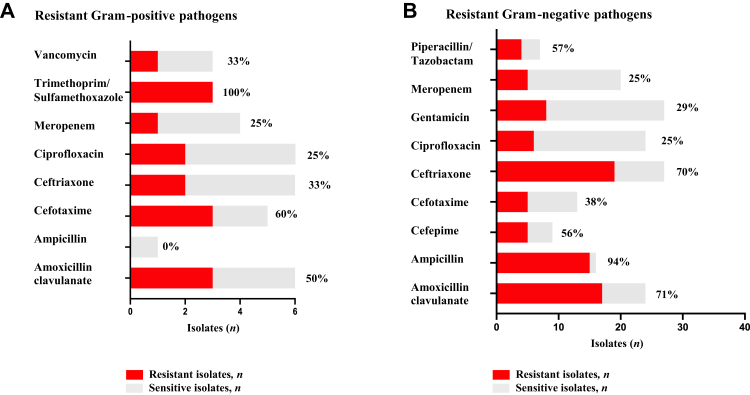
**Proportion of resistant bacterial pathogens isolated in people living with HIV with sepsis**. Bar graph showing frequency of detected Gram-positive (A) and Gram-negative (B) drug resistant bacteria in the subset of isolates with DST (n = number of resistant isolates; % = percent resistant isolates; Pseudomonas counted as resistant to Ampicillin, Ceftriaxone, Cefotaxime, Amoxicillin clavulanate, Trimethoprim/Sulfamethoxazole and Vancomycin regardless of in vitro DST results). DST = Drug sensitivity testing.

Drug susceptibility data from routine clinical care were available for 46 (33%) of 139 cultured non-*Mtb* bacterial isolates. We detected ceftriaxone resistance in 21 (64%) of 33 isolates for which the drug was tested (Fig. 2A and B and Supplementary Tables S3 and S4). We isolated *P. aeruginosa*, which is intrinsically resistant to ceftriaxone, in 5 (11%) of 46 cultures with available drug sensitivity, and in 4 (7%) of 55 non-*Mtb* bloodstream infections.

### Tuberculosis testing

**Fig. 3:**
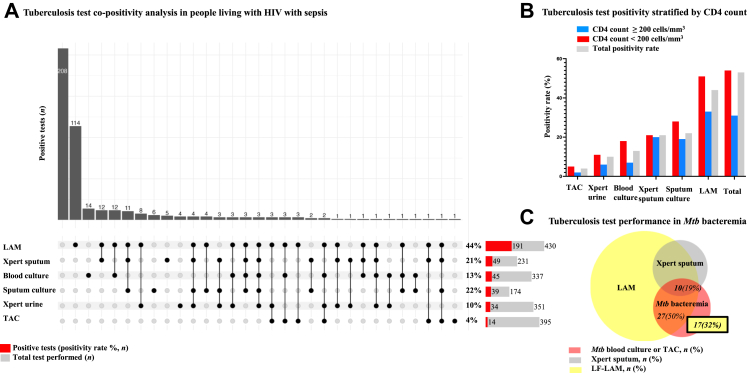
**Tuberculosis test diagnostic performance in people living with HIV with sepsis in sub-Saharan Africa**. (A) Upset plot showing intersection size of performed TB tests in all included trial participants with sepsis, and associated bar graph showing positivity rate for each test. N = intersection size. LAM = urine lateral flow lipoarabinomannan; Xpert = GeneXpert MTB/RIF or GeneXpert MTB/RIF Ultra; TAC = TaqMan Array Card PCR assay. (B) Rate of TB test positivity based on CD4 count. (C) Proportional Venn Diagram showing detection of *Mtb* bacteremia by LF-LAM and Xpert Sputum (n = number of participants; % = percent of all participants with *Mtb* bacteremia).

Of the 437 randomised participants 229 (52%) had a positive tuberculosis test result. Urine LF-LAM and *Mtb* blood cultures were the most common tests to be positive alone without another positive tuberculosis test (positive alone in 114 and 14 participants, respectively) (Fig. 3A and Table 4). There was no other common pattern of overlapping test positivity. LF-LAM was positive in 191 (44%) of 430 tests, sputum culture was positive in 39 (22%) of 174 tests, sputum GeneXpert MTB/RIF was positive in 49 (21%) of 231 tests, and urine GeneXpert MTB/RIF was positive in 34 (10%) of 351 tests. One MTB/RIF test revealed rifampin resistance and 5 had indeterminate results. All cultured *Mtb* was rifampin susceptible by phenotypic testing.

**Table 4:** TB test positivity rate stratified by CD4+ T-cell concentration among participants in a randomized clinical trial of early empiric anti-*Mycobacterium tuberculosis* therapy for sepsis in sub-Saharan Africa (ATLAS trial).

TB test	Total tested	Total resulted	Positive of resulted (%)	CD4 < 200 (%)	CD4 ≥ 200 (%)
TaqMan Array	426	395	4	5	2
Xpert MTB/RIF urine	351	351	10	11	6
*Mtb* blood culture	367	337	13	18	7
Xpert MTB/RIF sputum	231	231	21	21	20
*Mtb* sputum culture	184	174	22	28	19
Urine LF-LAM	430	430	44	51	33
All	437	435	53	54	31

All frequencies rounded to integer. LAM = urine lateral flow lipoarabinomannan; Gene/Xpert MTB/RIF or Gene/Xpert MTB/RIF Ultra; TAC = TaqMan Array Card PCR assay.

Of the 162 participants with a positive tuberculosis test and known CD4+ T-cell concentration, 103 (64%) had a CD4+ T-cell concentration <200 cells/mm^3^. Among participants with a CD4+ T-cell concentration <200 cells/mm^3^, the LF-LAM test positivity rate was 51% compared to 33% for those with ≥200 cells/mm^3^ (OR 0.47, 95% CI 0.30–0.77, p = 0.0023) and *Mtb* blood culture positivity was 18% compared to 7%, respectively (OR = 0.33, 95% CI 0.12–0.84, p = 0.02) (Fig. 3B and Supplementary Figures S4 and S5). Of the 54 *Mtb* bloodstream infections detected by culture or TAC, urine LF-LAM was positive in 27 (50%) and sputum GeneXpert MTB/RIF and urine LF-LAM were both positive in 10 (19%) instances. Therefore, rapid WHO-recommended tests missed 17 (32%) of *Mtb* bloodstream infections (Fig. 3C).

### Predicted improvement in sepsis antimicrobial coverage with expanded diagnostics and regimen composition

**Fig. 4:**
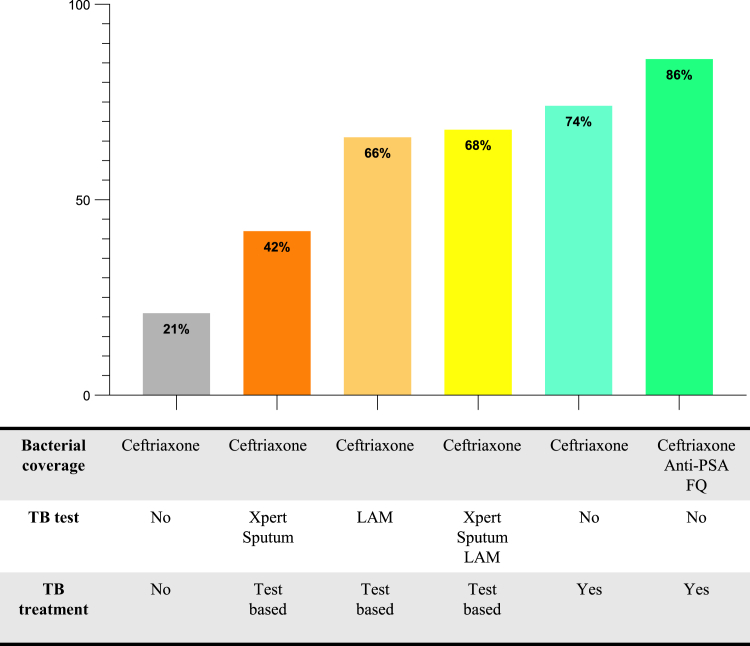
**Predicted antimicrobial coverage of people living with HIV with sepsis with expanded diagnostics and regimen composition**. Bar graph showing predicted antimicrobial coverage in PLWH with sepsis with ceftriaxone only, ceftriaxone and TB testing-based TB treatment, and expanded antimicrobial regimen with ceftriaxone, anti-PSA antibiotics and anti-*Mtb* antimicrobials. Xpert = GeneXpert MTB/RIF or GeneXpert MTB/RIF Ultra; LAM = urine lateral flow lipoarabinomannan; anti-PSA FQ = anti-pseudomonal fluoroquinolone.

Based on the observed frequency of ceftriaxone-resistant pathogens and the proportion of patients with *Mtb* infection that were missed by conventional WHO-recommended first-line diagnostics, a regimen consisting of only ceftriaxone without TB testing or anti-*Mtb* treatment would appropriately treat 21% of identified pathogens (Fig. 4 and Supplementary Table S5). In comparison, urine LF-LAM and sputum GeneXpert MTB/RIF testing-based early anti-TB therapy initiation, in combination with ceftriaxone treatment, would appropriately treat 68% of identified pathogens. An expanded spectrum antimicrobial regimen that included empirical anti-TB therapy and antibacterials active against ceftriaxone susceptible and resistant non-*Mtb* bacteria such as *P. aeruginosa*, would treat 86% of identified and plausibly causative sepsis pathogens (not accounting for *Cryptococcus* species excluded from the patient population, and possible pathogens of *Plasmodium* species).

### Clinical prediction of *Mtb* detected by one or more laboratory methods

**Fig. 5:**
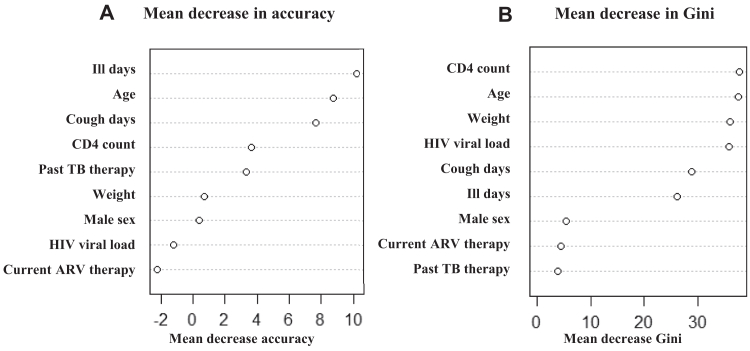
**Contribution of variables to random forest model of tuberculosis sepsis prediction**. Random forest plot analysis of clinical variables and tuberculosis positivity is showing the mean decrease in accuracy (A) and Gini (B) in the accuracy of prediction of tuberculosis infection with each variable. ARV = antiretroviral.

The baseline clinical variables in participants with and without positive TB tests are depicted in Table 5. Multivariable logistic regression analysis of these variables did not yield any significant predictors of TB tests positivity (Supplementary Table S6). From the random forest analysis, a greater number of ill days before presentation, younger age, a greater number of days of cough, lower CD4+ T-cell concentration, no prior anti-TB therapy, and male sex were associated with *Mtb* as a sepsis etiology. The mean decrease in accuracy and mean decrease in Gini are shown in Fig. 5 and Supplementary Table S7. However, the out-of-bag error rate was high at 41%, with accuracy and precision of 0.6 and 0.5, respectively. The recall was 0.6, and the F1-Score was 0.5. The Lasso regression model and XGBoost analysis results are shown in Supplementary Table S8 and Supplementary Figure S6, respectively.

**Table 5:** Baseline variables of participants with TB positive or TB negative test results in a randomized clinical trial of early empiric anti-*Mycobacterium tuberculosis* therapy for sepsis in sub-Saharan Africa (ATLAS trial).

Variable	*Mtb* positive	*Mtb* negative	Odds ratio	95% Confidence interval	p-value
Age (years), median (IQR)	40 (32–50)	43 (35–54)	**---**	**---**	**0.007**
Weight (kg), median (IQR)	48.5 (44–55)	50 (45–58)	**---**	**---**	**0.025**
Sex, male (%)	49	44	**---**	**---**	ns
Past TB treatment (%)	6	12	0.44	[0.22–0.88]	**0.02**
Ill days, *n*, median (IQR)	30 (14–60)	21 (14–56)	**---**	**---**	**0.005**
Cough days, *n*, median (IQR)	30 (14–30)	30 (17–45)	**---**	**---**	ns
CD4 cells/mm^3^, median (IQR)	90 (27–322)	138 (37–352)	**---**	**---**	ns
Current ART (%)	44	53	**---**	**---**	ns

All values rounded to integer except values lower than 1. Mann–Whitney test and odds-ratios were used for statistical analysis. p values < 0.05 were considered significant and are indicated in bold.

## Discussion

Unlike previous studies of sepsis etiology from Europe and North America where a causative sepsis pathogen is identified in only a minority of patients, in this study of PLWH in semi-urban settings of Tanzania and Uganda, we detected a plausible pathogen of sepsis in 69% of patients. *Mtb* was the most frequently detected pathogen of sepsis and accounted for 50% of bloodstream infections. *S. pneumoniae*, *S. aureus*, *Klebsiella* species*,* and *E. coli* were the most frequently isolated non-tuberculosis sepsis pathogens, and over 50% of the bacterial isolates tested were resistant to ceftriaxone. We also established that a greater number of ill-days before presentation, younger age, a greater number of days of cough, and low CD4+ T-cell concentration best indicated *Mtb* as a sepsis pathogen.

These findings are particularly concerning given that one in four critically ill patients in Africa has an infection, and infection and HIV are independently associated with increased mortality.^21^ The biologically plausible associations between clinical risk factors and tuberculosis were reassuring regarding the high prevalence of tuberculosis-sepsis identified in our study. Yet, none of the variables or the associated random forest model could confidently rule-in or rule-out tuberculosis sepsis, suggesting that tuberculosis sepsis cannot be predicted based on clinical variables alone in this critically ill population. Our findings help to inform the construction of a comprehensive empirical antimicrobial regimen for PLWH with sepsis in East Africa that may also be applicable to other tuberculosis-endemic settings.

While other observational studies have demonstrated that *Mtb* is the most common cause of sepsis in Africa,^6^^,^^9^^,^^14^^,^^22^ our study demonstrated a higher than previously reported prevalence of *Mtb.* This is likely related to our focus on a critically ill population, the rigorous testing for *Mtb,* which included mycobacterial blood culture and blood qPCR, in addition to conventional rapid diagnostics, and the high proportion of participants with a CD4+ T-cell concentration <200 cells/mm^3^ in whom LF-LAM is more sensitive and specific.^23^ Since our study was limited to East Africa, further studies in Africa and other TB-endemic regions are needed to determine if there are regional variations in sepsis etiology related to the proportion of adult PLWH.

WHO guidelines recommend initiating antituberculosis treatment to adult PLWH with critical illness when there is a high clinical suspicion for tuberculosis or a positive tuberculosis test including urine LF-LAM and sputum nuclear acid amplification test.^24^ However, our findings demonstrate that combined testing with sputum GeneXpert MTB/RIF and urine LF-LAM missed 32% of patients with *Mtb* bloodstream infection. Higher sensitivity LF-LAM designs and other novel rapid molecular diagnostics including those that test blood, may close this diagnostic gap. Limited autopsy studies of individuals with and without HIV in sub-Saharan Africa indicate that the prevalence of disseminated *Mtb* as a cause of death may be significantly higher than what can be detected during hospitalization.^25^ In sepsis treatment, effective antimicrobial therapy should start as soon as possible, as even hours of delay, which may be required for a tuberculosis test result to return, may lead to higher mortality.^26^, ^27^, ^28^ Our findings suggest that an immediate empirical antimicrobial regimen for sepsis in PLWH from tubercluosis-endemic regions should include antituberculosis treatment.

Empirical antimicrobial therapy in this population should additionally account for the high rate of predicted resistance to ceftriaxone among non-*Mtb* bacteria. The non-*Mtb* species distribution and resistance patterns found in this cohort were reflective of consistent trends in related populations across the continent, which show that Africa has some of the highest rates of antimicrobial resistance worldwide.^29^^,^^30^ Despite efforts to implement national action plans on antimicrobial resistance,^31^ many district hospitals in Africa, where PLWH with sepsis often seek care, do not routinely perform blood or urine cultures, or drug susceptibility testing. Instead, antibiotic selection is often influenced by cost and availability. In such a context, an empiric antimicrobial regimen for sepsis that includes an anti-pseudomonal fluoroquinolone would likely have activity against *Pseudomonas* infections, many Enterobacteriales that are resistant to ceftriaxone, and *Mtb*. Similarly, bacterial species such as methicillin resistant *S. aureus* and *Enterococcus* spp. could be targeted with linezolid which also treats *Mtb*. While specific combinations, companion drugs with ceftriaxone, routes of administration, doses, and duration would require comparative prospective study, our findings suggest that a regimen that targets *Mtb* and ceftriaxone-resistant bacteria would treat greater than 80% of all plausible pathogens of sepsis at our study hospitals.

Our study had several limitations. First, although we performed an extensive evaluation for TB, there is no “gold standard” for TB diagnosis in the critically ill given the low sensitivity of cultures and limited sensitivity and specificity of LF-LAM. Additionally, our bacterial blood culture positivity rates were low. This may be due to some participants being referred from rural clinics, where they may have received an antibacterial prior to transfer. We hypothesize this as a reason for the higher rate of bacterial bloodstream infection detected by qPCR compared to bacterial culture. Conversely, while we detected multiple urinary pathogens we cannot confirm they were causative organisms for sepsis. The study excluded those with positive serum CrAg at screening or by history prior to other comprehensive testing. CrAg positivity was low (4% of screened participants); however, given this exclusion criterion, we were unable determine the rate of cryptococcal co-infection with other putative pathogens of sepsis. Our study also did not include systematic drug susceptibility testing for each positive bacterial culture, which instead was only performed at the treating clinician's discretion. While there is a risk of overestimating ceftriaxone resistance if clinicians preferentially tested samples from patients that were not responding to standard treatments, our data are consistent with prior studies showing high rates of ceftriaxone-resistant Gram-negative bacteria regionally.^32^^,^^33^ Finally, we detected region-specific differences in *Mtb* sepsis and bacterial sepsis prevalence between Tanzania and Uganda, which highlights the need for more studies investigating region-specific sepsis etiology and resistance patters.

In conclusion, in a large cohort of PLWH with sepsis from representative referral hospitals in Tanzania and Uganda that reflect regionally common clinical settings and practice patterns, we conducted a comprehensive analysis of sepsis etiology. An enhanced spectrum antimicrobial therapy that targets *Mtb* and other ceftriaxone-resistant bacterial pathogens should be considered for empirical treatment of sepsis in PLWH in similar populations in East Africa and potentially other areas with high HIV and TB prevalence.

## Contributors

E.O. and L.A. conceptualized the manuscript, reviewed, analyzed, and interpreted data, and wrote the manuscript. L.A. also supervised molecular testing at the Uganda study site, and E.O. performed literature search and review and visualized the data. The first co-authors contributed equally. M.N. and P.R. analyzed data, visualized data, and contributed to reviewing the manuscript. E.O. and M.N. had access to and verified the underlying study data. C.C.M., C.M., S.M., D.B., and S.H. designed the ATLAS trial, acquired funding, and served as co-investigators for the trial. They also reviewed the data and the manuscript draft. C.C.M. supervised the manuscript data collection, presentation, analysis, and interpretation. B.S., M.S., E.N., G.G., A.G., S.J., A.R., F.M., J.K., O.A., D.O., and D.M. recruited participants, collected and analyzed data, and edited the manuscript draft. J.L. contributed to supervising molecular testing, data analysis, and reviewing the manuscript. B.M. contributed to molecular testing and edited the manuscript. T.T. led LF-LAM testing during the ATLAS trial and reviewed and edited the data and manuscript.

## Data sharing Statement

Metadata and standard operating procedures are available upon request to the corresponding author with publication and following approval of the proposal by the trial executive committee.

## Declaration of interests

All authors declare no competing interests.

## Appendix A Supplementary data

Supplementary Tables and Figures

Translated Abstracts in Runyankole

Translated Abstracts in Swahili
