# Supplementary material for: Aetiology of sepsis in adults living with HIV in East Africa: a secondary analysis of an open-label, multicentre, randomised, controlled phase 3 trial
Source: eClinicalMedicine. 2026 Jan 28;92:103719. doi: 10.1016/j.eclinm.2025.103719 (PMC12947645; doi:10.1016/j.eclinm.2025.103719)
Supplement: Supplementary Tables and Figures [file mmc1.pdf]

Supplementary Table 1: TAC Assay targets used to evaluate for bloodstream infection in participants a randomized clinical trial of early empiric anti-*Mycobacterium tuberculosis* therapy for sepsis in sub-Saharan Africa (ATLAS trial)

Cytomegalovirus	*Listeria monocytogenes*
Acinetobacter baumannii	*Mycobacterium avium* complex
Bartonella species	Marburg
Brucella species	*Mycobacterium tuberculosis*
Bundibugyo	*Neisseria meningitidis*
Crimean-Congo Hemorrhagic Fever	Nipah
Chikungunya	O'nyong'nyong virus
Coxiella burnetii	*Plasmodium* species
Cryptococcus neoformans	*Pseudomonas aeruginosa*
Dengue	*Rickettsia* species
Ebola	Rift Valley Fever
Enterovirus	*Salmonella* species
Escherichia coli	*Salmonella Typhi*
Group A *Streptococcus*	*Staphylococcus aureus*
Group B *Streptococcus*	*Streptococcus pneumoniae*
Hantavirus (Hantaan and Seoul)	Sudan Ebolavirus
Hemophilus influenzae	*Toxoplasma gondii*
Hepatitis E	*Trypanosoma brucei*
Histoplasma species	West Nile Virus
Klebsiella pneumoniae	Yellow Fever
Leishmania species	*Yersinia pestis*
Leptospira species	

Supplementary Table 2: Infectious etiology divided by clinical site.

**Site**	**Tanzania**	**Uganda**	**Odds ratio**	**95% Confidence interval**	**p-value**
Participants, *n*	247	190			
Baseline characteristics					
Sex, female, *n* (%)	132 (54)	101 (53)	**---**	**---**	ns
Age, median (IQR)	44 (35-53)	40 (30-49)	**---**	**---**	**0.0001**
CD4 count, median (IQR)	124 (29-321)	119 (28-350)	**---**	**---**	ns
ART, *n* (%)	118 (48)	91 (48)	**---**	**---**	ns
Sepsis etiology					
TB positive, *n* patients (% all)	148 (60)	81 (43)	2.01	[1.37-2.95]	**0.0004**
Bacterial pathogens, *n* patients (% all)	54 (22)	85 (45)	0.35	[0.23-0.52]	**<0.0001**
Bacterial BSI, *n* patients (% all)	24 (10)	31 (16)	0.55	[0.31- 0.98]	**0.0412**
TB BSI by culture, *n* patients (% resulted)	20/171 (12)	25/166 (15)	**---**	**---**	
TB BSI by TAC, *n* patients (% resulted)	7/217 (3)	7/178 (4)	**---**	**---**	

Mann-Whitney and odds ratio were used for statistical analysis of differences between the two sites. Odds ratios are calculated for Tanzania site. TB=tuberculosis, BSI=blood stream infection.

Supplementary Table 3: Antimicrobial resistance patterns of Gram-positive bacteria from participants in a randomized clinical trial of early empiric anti-*Mycobacterium tuberculosis* therapy for sepsis in sub-Saharan Africa (ATLAS trial)

Resistant Gram-positive isolates, n/tested	Amoxicillin clavulanate	Ampicillin	Cefotaxime	Ceftriaxone	Ciprofloxacin	Meropenem	Trimethoprim/ Sulfamethoxazole	Vancomycin
**Blood**								
Streptococcus pneumoniae	1/1			0/1		0/1		
Staphylococcus aureus	0/1	0/1	1/1	0/1	1/3	1/3		1/2*
**Urine**								
Enterococcus species	0/1		0/1	0/1	1/2			0/1
Staphylococcus aureus	2/3		2/3	2/3	0/3		3/3	
**Total**	**3/6**	**0/1**	**3/5**	**2/6**	**2/8**	**1/4**	**3/3**	**1/3***
**Total %**	**50%**	**0**	**60%**	**33%**	**25%**	**25%**	**100%**	**33%***

*Intermediate resistance

Supplementary Table 4: Antimicrobial resistance patterns Gram-negative bacteria among participants in a randomized clinical trial of early empiric anti-*Mycobacterium tuberculosis* therapy for sepsis in sub-Saharan Africa (ATLAS trial)

Resistant Gram-negative isolates, n/tested	Amoxicillin clavulanate	Ampicillin	Cefepime	Cefotaxime	Ceftriaxone	Ciprofloxacin	Gentamycin	Meropenem	Piperacillin/Tazobactam
**Blood**									
Citrobacter species	0/1	0/1	0/1	0/1	0/1		0/1	0/1	0/1
Klebsiella pneumoniae				1/1	1/1			0/1	
Pseudomonas aeruginosa	3/3*	3/3*		1/1*	3/3*	0/1	0/3	0/1	
Salmonella species	0/1	1/1	0/1	0/1	0/1	0/1	1/1	0/1	
**Urine**									
Acinetobacter baumanii			1/1		1/1		0/1	1/1	
Citrobacter species	1/1	1/1	1/1		1/1	0/1	0/1	0/1	
Enterobacter species	1/1	1/1	1/1		1/1		1/1	1/1	1/1
Proteus species	0/2	1/1		0/2	1/2	0/2	1/3	0/1	
Pseudomonas aeruginosa	1/1*	1/1*			2/2*		1/1**		1/1
Klebsiella pneumoniae	5/7	3/3	2/2	2/5	4/8	3/8	1/8	1/3	1/1
Escherichia coli	6/7	4/4	1/2	1/2	5/6	3/11	3/7**	2/9	1/3
**Total**	**17/24**	**15/16**	**5/9**	**5/13**	**19/27**	**6/24**	**8/27**	**5/20/0**	**4/7**
**Total (%)**	**71%**	**94%**	**56%**	**38%**	**70%**	**25%**	**29%**	**25%**	**57%**

*Inherent resistance **Intermediate resistance

Supplementary Table 5: Predicted bacterial and TB sepsis coverage according to testing and treatment regimens among participants in a randomized clinical trial of early empiric anti-*Mycobacterium tuberculosis* therapy for sepsis in sub-Saharan Africa (ATLAS trial)

**Testing and treatment Regimen**	Gram positive resistant isolates (%)	Gram negative resistant isolates (%)	Gram positive resistant isolates (weighted % participants)	Gram negative resistant isolates (weighted % participants)	TB infection not covered* (weighted % participants)	Other sepsis causing organisms** not covered (weighted % participants)	Organisms not treated (% participants)
Ceftriaxone only; no TB testing or treatment	33	70	4	18	53	4	79
Ceftriaxone + Xpert sputum	33	70	4	18	32	4	58
Ceftriaxone + LAM testing	33	70	4	18	8	4	34
Ceftriaxone + LAM + Xpert sputum testing	33	70	4	18	6	4	32
Ceftriaxone + empiric anti-*Mtb* therapy	33	70	4	18	0	4	26
Ceftriaxone + anti-PSA fluoroquinolones + empiric anti-*Mtb* therapy	25	25	3	7	0	4	14

*Not accounting for co-infections and test co-positivity

***Cytomegalovirus* and *Plasmodium* excluded

Xpert=*Mtb*/RIF Gene/Xpert; LAM=urine lateral flow lipoarabinomannan; anti-PSA fluroquinolones=anti-pseudomonal fluroquinolones; TB=tuberculosis; BSI=blood stream infection.

Supplementary Table 6: Logistic regression analysis of multiple variables for TB predictors.

	Estimate	Standard Error	Z value	p-value
Age	-0.02	0.01	-1.2	0.2
Weight	-0.03	0.02	-1.4	0.14
Male sex	0.47	0.43	1.09	0.27
Past TB treatment	-0.37	0.84	-0.43	0.66
Ill days	-0.0006	0.008	-0.08	0.93
Cough days	-0.001	0.008	-0.12	0.9
CD4 count	-0.0005	0.001	-0.36	0.71
Current ART	0.3	0.45	0.68	0.49
HIV viral load	2.61E-08	1.44E-07	0.18	0.85

TB=tuberculosis; ART=Antiretroviral Therapy

Supplementary Table 7: Mean decrease in accuracy and Gini in a random forest model of TB sepsis using data acquired from participants in a randomized clinical trial of early empiric anti-*Mycobacterium tuberculosis* therapy for sepsis in sub-Saharan Africa (ATLAS trial)

Variable	Mean Decrease in Accuracy	Mean Decrease in Gini
Age, years (median)	8.7	37.5
Weight, kg (mean)	0.7	36.1
Male sex (%)	0.4	5.4
Past TB treatment (%)	3.3	3.9
Ill days (median)	10.1	26.2
Cough days (median)	7.7	28.9
CD4 cells/mm^3 (median)	3.6	37.7
Current ART (%)	-2.2	4.4
HIV viral load (copies/ML)	-1.2	35.9

TB=tuberculosis; ART=Antiretroviral Therapy

Supplementary Table 8: Estimated coefficients for TB predictors (LASSO regression model)

Variable	Estimated coefficients
Intercept	2.104
Age, years (median)	-0.0136
Weight, kg (mean)	-0.0212
Male sex (%)	-0.244
Past TB treatment (%)	-0.6222
Ill days (median)	0.0023
Cough days (median)	-0.0056
CD4 cells/mm^3 (median)	0
Current ART (%)	-0.1203
HIV viral load (copies/ML)	0

TB=tuberculosis; ART=Antiretroviral Therapy

Supplementary Figure 1: ATLAS randomized controlled trial profile

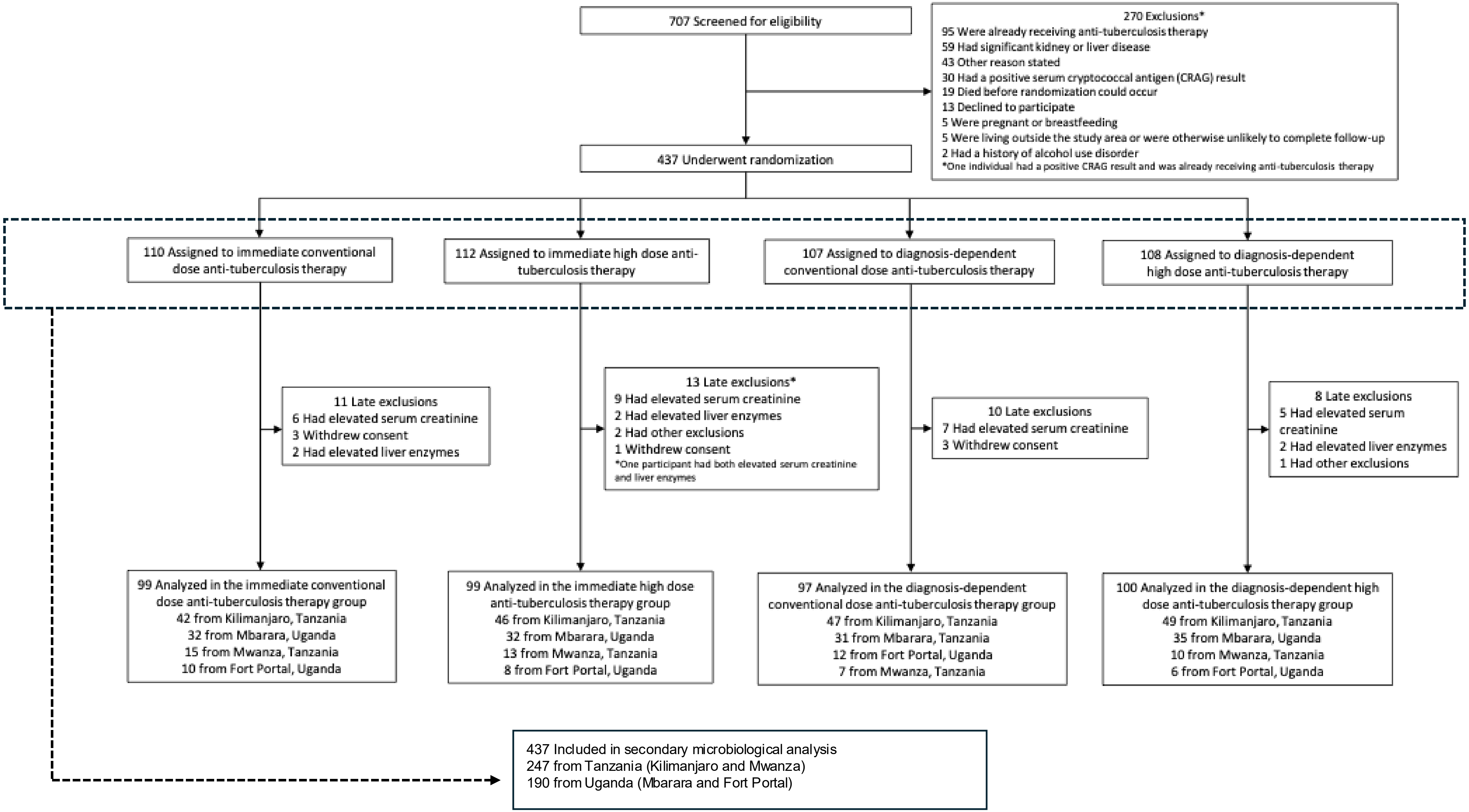

Supplementary figure 1: CONSORT diagram of ATLAS randomized controlled trial.

Supplementary Figure 2: Isolated sepsis pathogens in people living with HIV with sepsis

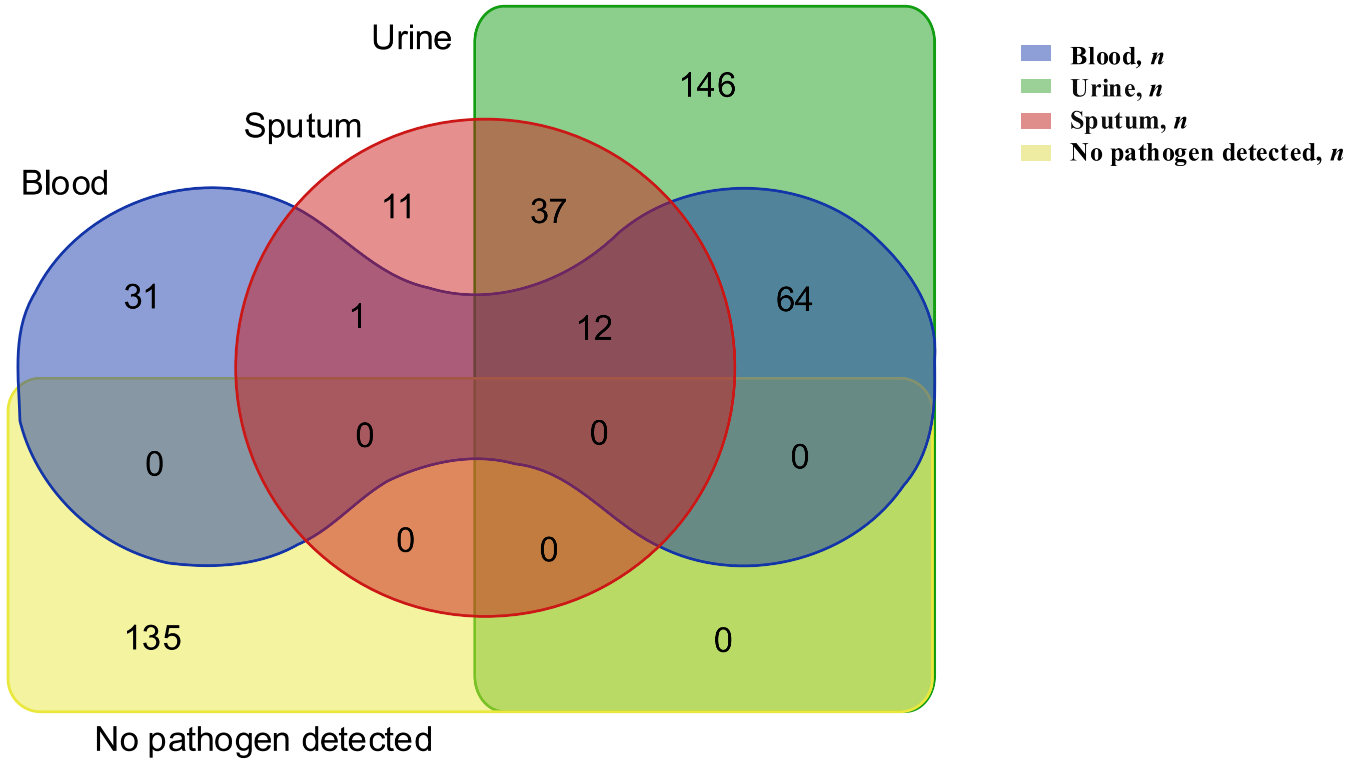

Supplementary figure 2: Venn Diagram showing *n* of positive tests per all participants, divided by specimen. Excluding Cytomegalovirus and *Plasmodium*.

Supplementary Figure 3: Cytomegalovirus and *Plasmodium* co-infection in people living with HIV with sepsis

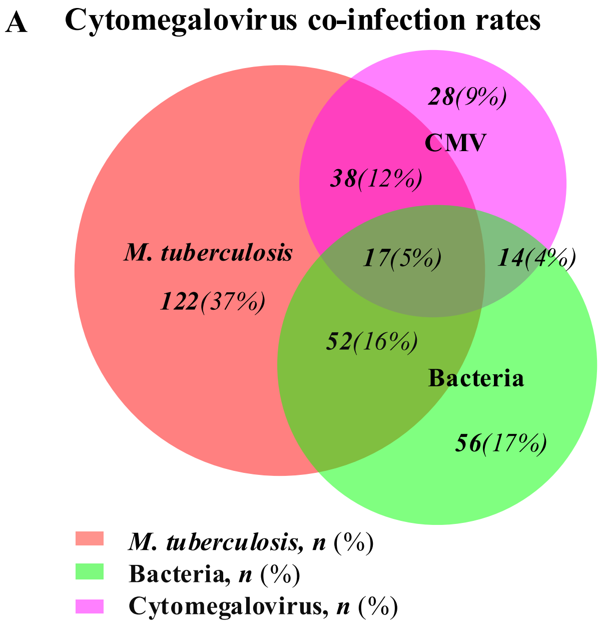

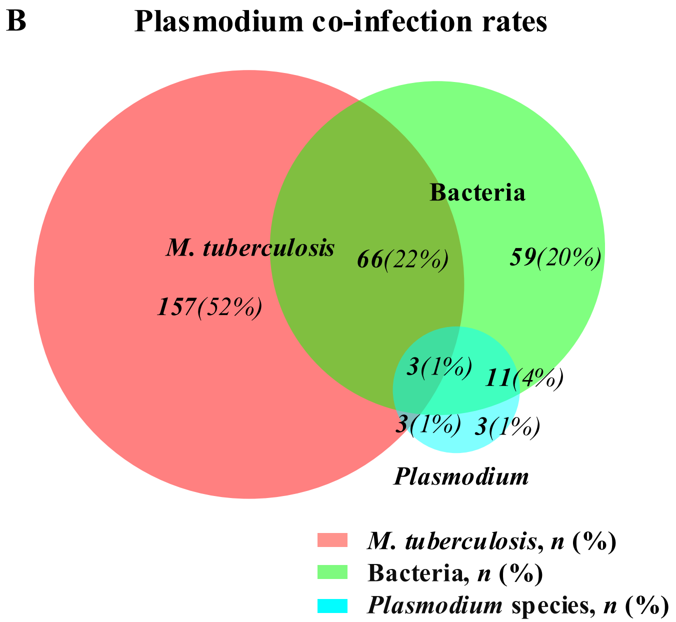

Supplementary figure 3: Proportional Venn Diagram showing rates of co-infections with CMV (A) and *Plasmodium* (B) (n=number of participants with pathogen detected, %=percentage of all participants included). CMV=Cytomegalovirus.

Supplementary Figure 4: Tuberculosis test co-positivity analysis in people living with HIV with sepsis and CD4 count < 200 cells/mm^3

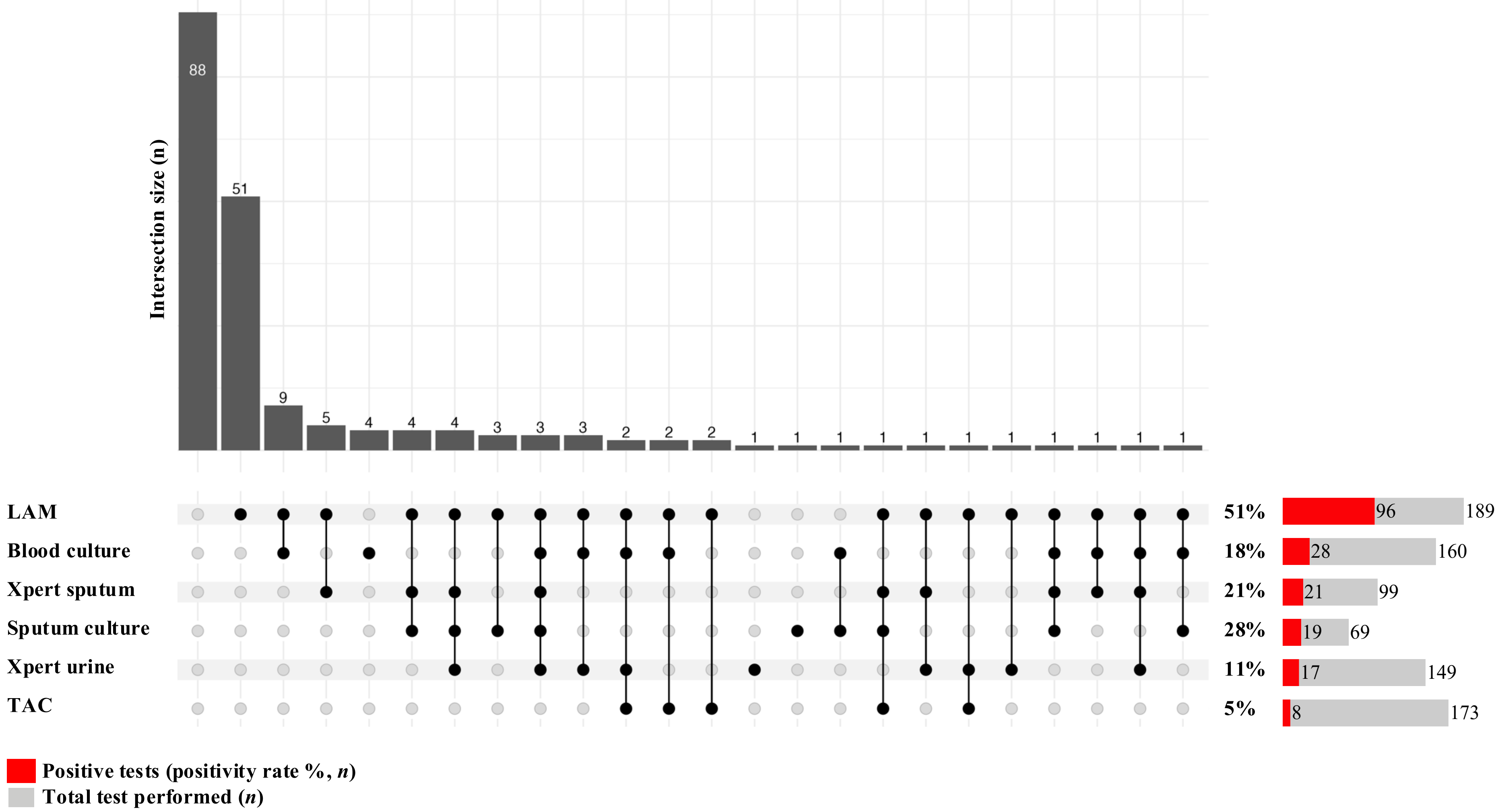

Supplementary figure 4: Upset plot showing intersection size of performed tuberculosis tests in all included trial participants with sepsis and CD4 count < 200 cells/mm^3, and associated bar graph showing positivity rate for each test. *N*=intersection size. LAM=urine lateral flow lipoarabinomannan; Xpert= GeneXpert *Mtb*/RIF or GeneXpert *Mtb*/RIF Ultra; TAC=TaqMan Array Card PCR assay.

Supplementary Figure 5: Tuberculosis test co-positivity analysis in people living with HIV with sepsis and CD4 count ≥200 cells/mm³

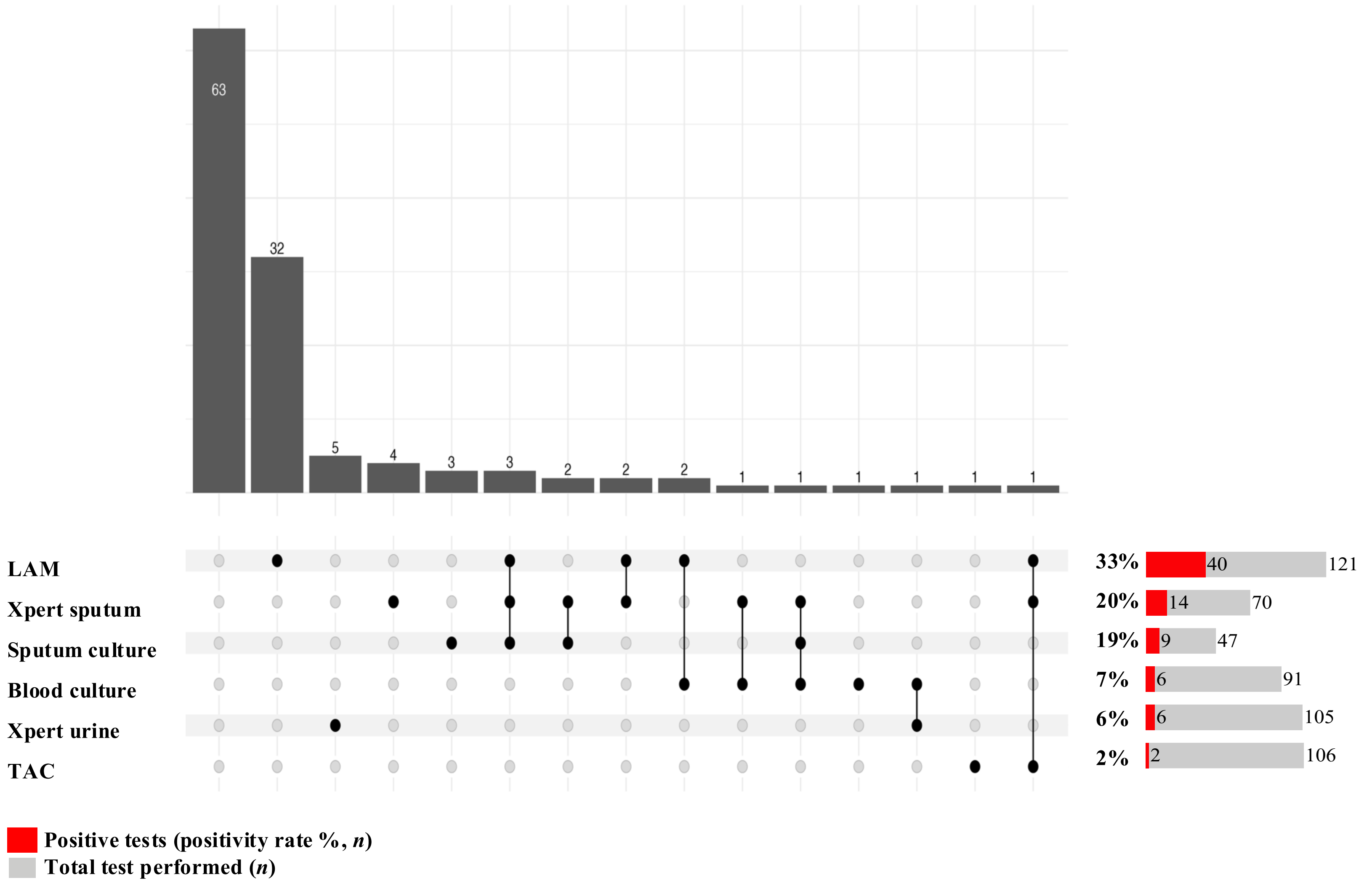

Supplementary figure 5: Upset plot showing intersection size of performed tuberculosis tests in all included trial participants with sepsis and CD4 count ≥ 200 cells/mm³, and associated bar graph showing positivity rate for each test. *N*=intersection size. LAM=urine lateral flow lipoarabinomannan; Xpert= GeneXpert *Mtb*/RIF or GeneXpert *Mtb*/RIF Ultra; TAC=TaqMan Array Card PCR assay.

Supplementary Figure 6: Predictors of tuberculosis positivity (XGBoost Model)

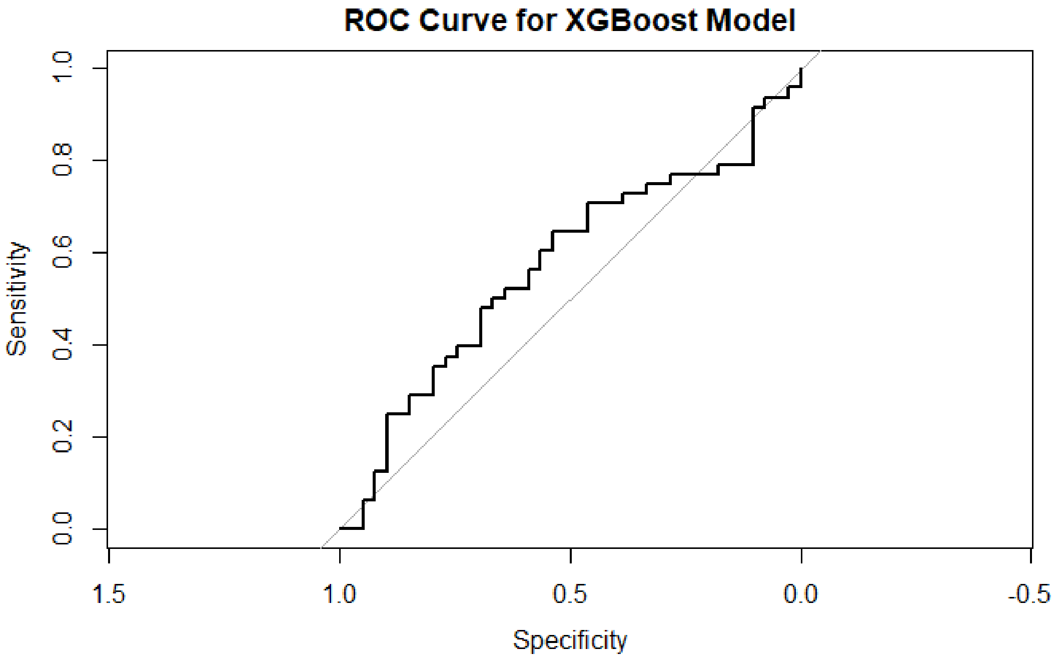

Supplementary figure 6: Receiver operating characteristic (ROC) curve for the XGBoost model predicting tuberculosis infection. The XGBoost model achieved an area under the curve (AUC) of 0.5694, indicating modest discriminatory ability to distinguish individuals with tuberculosis infection from those without. The model included age, weight, sex, prior tuberculosis treatment, duration of illness, duration of cough, CD4 cell count, current antiretroviral therapy use, and HIV viral load.
