# Supplementary material for: Aetiology of sepsis in adults living with HIV in East Africa: a secondary analysis of an open-label, multicentre, randomised, controlled phase 3 trial
Source: eClinicalMedicine. 2026 Jan 28;92:103719. doi: 10.1016/j.eclinm.2025.103719 (PMC12947645; doi:10.1016/j.eclinm.2025.103719)
Supplement: Translated Abstracts in Runyankole [file mmc2.docx]

***The following translations in Runyankole were submitted by the authors and we reproduce them as supplied. They have not been peer reviewed. Our editorial processes have only been applied to the original abstract in English, which should serve as reference for this manuscript.***

**Omubugufu**

**Obutandikiro** Oburwiire bw’okutabuka kw’omubiri bukuruga aha bukoko ningashi oburofa bw’omushagama omubantu abarikutuura n’akakooko (TB) ka sirimu (PLWH) omu mahanga ga murugwa izooba bwa Afirika nibwiita munonga. Aharurengo rw’ekyanga eki, ebirikureeta oburwiire obu tibiketegyerezibwe_gye. Tukakora okucoondooza turikwejunitsa ebihandiiko ebyabeire biriho ebitwatungire kuruga omukugyezetsa anti-*Mycobacterium tuberculosis* (*Mtb*) omubazi gw’oburofa bw’omushagama omuri Tanzania na Uganda (ATLAS trial)

**Enkora/Ebyakozirwe** Okucoondooza oku, okw’idaara lya kashatu eri kugyezesa omubaazi, kukakorerwa aha marwariro abiri amahango omuri Tanzania na Uganda. Abayejumbiiremu bakaba bari abantu abakweshariramu, abarengize emyaka 18, abarikutuura na TB ka siriimu, baheirwe ebitanda ahabw’endwaara ezeine akakwaate n’oburofa bw’omushagama hamwe n’okukyeberwa bakashangwa ebicweeka byaabo by’omunda bitarikukoragye (qSOFA) ≥2. Tukatwaaza tuti: (1) Obujanjabi bwa TB obwa bureijo obwaho-naho neinga kurugirira ebyaruga omukukyebera hamwe na: (2) Ekipiimo ky’omubazi kyamaani neinga ekyabureijo eky’emibazi y’a TB. Okukyebera ebirikureeta oburofa omushagama kukaba kurimu okukyebera obukooko bw’omushagama hamwe n’enkari, endagabutonde (qPCR) kuruga omushagama, GeneXpert MTB/RIF Ultra-eya oburwaire bwa TB, omukikororo hamwe n’enkari. Lipoarabinomannan (LF-LAM) ey’enkari, hamwe n’okuteeka ebikororo hamwe n’eshagama (Mtb cultures). Tukwejunitsa okukyebera kwemiringo myingi kugyezaho kwetegyereza ebirikuteberezibwa ahari *Mtb* nka bimwe ebirikureeta oburofa bw’omushagama. Okucoondooza oku kukahandikisibwa kandi enamba yakwo ni NCT04618198.

**Ebyarugiremu** Kuruga ebiro bitaano by’okweezi kw’okubanza kw’omwaka 2022 kuhika ebiro 9 by’okwikumi-n’ebiri omwaaka 2024, tukatorana abantu 437 kuheebwa doozi erikukarataho omumaani hamwe na/ neinga doozi eyamaani munonga eya TB. *Mtb* niko kakooko akasingireyo kukwaata, kakashangwa omu bantu 229 (52%) ahari 437 ababeire bejumbiiremu, hamwe n’omuri 54 (50%) ahari 108 ababeire beine oburofa bw’omushagama. Okukyebera enkari LF-LAM hamwe n’ekikororo GeneXpert MTB/RIF tikurabatsize kukwaata oburofa bw’omushagama obwa *Mtb* omubantu 17 (32%) ahari 54. Obukooko obwa non-mycobacterial bacteria obwasingireyo kukwaatwa bukaba buri obw’ekika kya Klebsiella hamwe na Escherichia coli, obwashangirwe omubantu 39 (9%) hamwe na 33 (8%) ahari 437 abebeire bejumbiiremu. Abi emibiri yaabo yabeire negaya ceftriaxone bakaba bari 21 (64%) ahari 33 ababeire bateirwe aharubaju. Omunkora ey’okugyeragyeranitsa erimu okuteebereza (accuracy: 0.6; precision: 0.5; recall: 0.6; F1-score: 0.5), kikashangwa ngu ekirikusingayo kwoorekagye *Mtb* nk’oburofa bw’omushagama kikaba kiine enaamba/ omuhendo muhango omubiro obu erikuba etakorekire bubonero (MDA 10.1), omumyaaka mito (MDA 8.7), omukugira orukororo kuheza obwiire bureingwa (MDA 7.7), hamwe na low CD4+ T-cell concentration (MDA 3.7).

**Okushoborora** *Mtb* nibwo bukooko obwashangirwe burikukizayo okurwaaza hamwe n’oburofa bw’omushagama kandi bukaba burikukira kutakwatwa bwakyeberwa orikwejunitsa ekyebera eya bureijo. Kandi nabwo tukashanga ngu hariho okukanya kw’obukokoko obutari bwa TB haza burikugaya Ceftriaxone omushagama hamwe n’enkari. Bimwe ahabyatutiire aha mukono omukucoondooza oku bika birimu okutejumbiramu kw’abo abeine omuraramu gwa cryptococcal, okutakyebera bureijo obukooko oburikugaya emibazi, hamwe n’entanitso eyakuba nebaho omubyanga bitarikushushana ahabiri kurofahaza eshagama n’okubirikugaya emibazi.
