# Supplementary material for: Aetiology of sepsis in adults living with HIV in East Africa: a secondary analysis of an open-label, multicentre, randomised, controlled phase 3 trial
Source: eClinicalMedicine. 2026 Jan 28;92:103719. doi: 10.1016/j.eclinm.2025.103719 (PMC12947645; doi:10.1016/j.eclinm.2025.103719)
Supplement: Translated Abstracts in Swahili [file mmc3.docx]

***The following translations in Swahili were submitted by the authors and we reproduce them as supplied. They have not been peer reviewed. Our editorial processes have only been applied to the original abstract in English, which should serve as reference for this manuscript.***

**Muhtasari**

**Utangulizi**: Sepsisi kwa watu wanaoishi na VVU (PLWH) ina kiwango kikubwa cha vifo katika Afrika Mashariki. Katikaukanda wa Africa, chanzo cha sepsis bado hakijaeleweka kikamilifu. Tulifanya uchambuzi wa data za kimaabara za kimaikrobiolojia zilizopatikana kutoka kwenye utafiti wa kitabibu wa majaribio (randomised clinical trial) wa tiba ya mapema dhidi ya bakteria wa kifua kikuu (Mtb) kwa wagonjwa wa sepsis (ATLAS) nchini Tanzania na Uganda.

**Mbinu:** Tunaonesha uchambuzi wa ziada uliowekwa mapema, wa utafiti wa kitabibu wa awamu ya tatu; wa wazi, wenye vituo vingi, na wa majaribio uliodhibitiwa, uliofanyika katika hospitali nne za rufaa za mikoa nchini Tanzania na Uganda. Washiriki walikuwa watu wazima wanaoishi na VVU waliolazwa hospitalini kwa mashaka ya maambukizi na alama ya *modified quick sepsis-related organ failure assessment (qSOFA)* ya 2 au zaidi.; ikimaanisha kuwa mgonjwa alikuwa na dalili kuu mbili au zaidi zinazohusiana na kushindwa kwa viungo kutokana na sépsis (qSOFA) ≥2. Washiriki waligawanywa kwa nasubi katika makundi mawili; (1) Kupokea tiba ya kifua kikuu (*antituberculosis therapy*) mara moja au kulingana na matokeo ya uchunguzi (diagnosis-dependent), na (2) Kupokea tiba ya kifua kikuu yenye dozi kubwa (high-dose) au dozi ya kawaida (conventional-dose). Vipimo vya kubaini chanzo cha sepsis vilijumuisha uchunguzi wa damu na mkojo kwa vijidudu (bacterial blood and urine cultures), multi-pathogen *qPCR* kutoka kwenye damu, GeneXpert MTB/RIF Ultra kutoka kwenye makohozi na mkojo, Vipimo vya mkojo kwa lipoarabinomannan (LF-LAM), na Vipimo vya Mtb kutoka kwenye makohozi na damu. Tulitumia uchambuzi wa *multivariable logistic regression* na *random forest analysis* kubaini vigezo vilivyotabiri uwepo wa Mtb kama chanzo cha sepsis. Utafiti huu umesajiliwa katika ClinicalTrials.gov, nambari ya usajili NCT04618198.

**Matokeo:** Kuanzia tarehe 5 Januari, 2022 hadi tarehe 9 Desemba 2024, tulihusisha washiriki 437 kupokea matibabu ya kifua kukuu ya haraka na/au yenye dozi ya kiwango cha juu. Vimelea vya kifua kikuu (Mtb)) ndivyo vilivyopatikana kwa wingi zaidi, ikigunduliwa kwa washiriki 229 (52%) kati ya washiriki 437, na katika 54 (50%) ya washiriki 108 walio na maambukizi ya damu. Upimaji wa mkojo kwa LF-LAM na makohozi kwa GeneXpert MTB/RIF haukuweza kubaini maambukizi ya damu ya Mtb kwa 17 (32%) kati ya wale 54 wenye maambukizi hayo. Bakteria wasiokuwa wa mycobacterial waliopatikana kwa wingi zaidi walikuwa Klebsiella species (washiriki 39, sawa na 9%) na Escherichia coli (washiriki 33, sawa na 8%). Upinzani dhidi ya ceftriaxone ulionekana katika 21 (64%) kati ya sampuli 33 za bakteria zilizopatikana. Katika muundo wa ubashiri wa msitu nasibu (*random forest prediction model*) (wenye usahihi wa jumla wa 0.6; usahihi wa utambuzi: 0.5; kumbukumbu: 0.6; alama ya F1:0.5), viashiria bora vya Mtb kama chanzo cha sepsis vilikuwa: idadi kubwa ya siku za ugonjwa kabla ya kufika hospitalini (MDA 10.1), umri mdogo (MDA 8.7), mda mrefu wa kikohozi (MDA7.7) na kiwango cha chini cha seli za kinga aina ya CD4+ T (MDA 3.7).

**Ufafanuzi:** Mtb ilikuwa kimelea kinachosababisha sepsis na maambukizi ya damu kwa kiwango kikubwa zaidi, na mara nyingi haikubainika kupitia vipimo vya haraka vya kawaida. Pia tulibaini uwepo wa kiwango cha juu cha vijidudu visivyo vya mycobacterial ambavyo vinaonyesha usugu dhidi ya ceftriaxone katika uchunguzi wa damu na mkojo. Mapungufu ya utafiti huu ni pamoja na kuwatenga washiriki waliokuwa na matokeo chanya ya *cryptococcal antigen*, kutofanyika kwa vipimo vya usugu wa dawa kwa utaratibu maalumu, pamoja na uwezekano wa kuwepo kwa tofauti za kieneo katika chanzo cha sepsis na muundo wa usugu wa vijidudu.
